# Visualizing the trigeminovagal complex in the human medulla by combining *ex-vivo* ultra-high resolution structural MRI and polarized light imaging microscopy

**DOI:** 10.1038/s41598-019-47855-5

**Published:** 2019-08-05

**Authors:** D. J. H. A. Henssen, B. Derks, M. van Doorn, N. C. Verhoogt, P. Staats, K. Vissers, A. M. Van Cappellen van Walsum

**Affiliations:** 1 Department of Anatomy, Donders Institute for Brain, Cognition & Behavior, Radboud university medical center, Nijmegen, The Netherlands; 2 Department of Radiology and Nuclear Medicine, Radboud university medical center, Nijmegen, The Netherlands; 3 Premier Pain Centers, Shrewsbury, New Jersey United States of America; 4 Department of Anesthesiology, Pain and Palliative Medicine, Radboud university medical center, Nijmegen, The Netherlands

**Keywords:** Anatomy, Brain, Brain

## Abstract

A trigeminovagal complex, as described in some animals, could help to explain the effect of vagus nerve stimulation as a treatment for headache disorders. However, the existence of a trigeminovagal complex in humans remains unclear. This study, therefore investigated the existence of the trigeminovagal complex in humans. One post-mortem human brainstem was scanned at 11.7T to obtain structural (T1-weighted) and diffusion magnetic resonance images ((d)MR images). Post-processing of dMRI data provided track density imaging (TDI) maps to investigate white matter at a smaller resolution than the imaging resolution. To evaluate the reconstructed tracts, the MR-scanned brainstem and three additional brainstems were sectioned for polarized light imaging (PLI) microscopy. T1-weighted images showed hyperintense vagus medullar striae, coursing towards the dorsomedial aspect of the medulla. dMRI-, TDI- and PLI-images showed these striae to intersect the trigeminal spinal tract (*sp5*) in the lateral medulla. In addition, PLI images showed that a minority of vagus fibers separated from the vagus trajectory and joined the trigeminal spinal nucleus (Sp5) and the *sp5*. The course of the vagus tract in the rostral medulla was demonstrated in this study. This study shows that the trigeminal- and vagus systems interconnect anatomically at the level of the rostral medulla where the vagus fibers intersect with the Sp5 and *sp5*. Physiological and clinical utility of this newly identified interconnection is a topic for further research.

## Introduction

Primary headache disorders are one of the most common disorders in neurology and cause substantial levels of disability globally^1,2^. Although the exact pathophysiological mechanisms that cause migraine and cluster headaches remain elusive, sensitization of the trigeminal nerve is thought to play an important role in primary headache disorders. For example, activation of the trigeminovascular system, with or without trigeminal autonomic features is thought to be the major mechanism that underlies both cluster headache and migraine attacks^3,4^. Furthermore, noxious stimulation of the meninges surrounding the brain and the dura mater in particular, or dural blood vessels (i.e. the superior sagittal sinus and the middle meningeal artery and other large cerebral arteries), can induce pain similar to that experienced when a person is suffering from a migraine or cluster headache attack^5–7^. Based on these mechanisms, modulation of the trigeminal nerve can be regarded as an experimental treatment option for both migraine and cluster headaches. For example, invasive neuromodulation of the sphenopalatine ganglion was found to be a promising treatment option for patients suffering from intractable migraine and cluster headaches^8–10^. However, non-invasive methods to treat primary headache disorders are available, as well and must be regarded as promising adjunct treatment options with excellent safety properties^10,11^. Conventionally, stimulating the vagus nerve (i.e. vagus nerve stimulation (VNS)) in the neck, non-invasively is referred to as non-invasive vagus nerve stimulation (nVNS), whereas transcutaneous vagus nerve stimulation (tVNS) is referred to when the auricular branch of the vagus nerve is targeted. In recent investigations, nVNS has been reported to be superior to sham treatment in treating migraine^12^ and episodic cluster headache^13^. In addition, tVNS was found to be a safe and effective treatment of chronic migraine in one study^14^. Although effective, well-tolerated and safe as a treatment of primary headache disorders, the exact neuroscientific underpinnings of VNS need to be further elucidated. Based on the clinical observations that VNS can influence pain presentation in primary headache disorders, our group hypothesized the existence of an interconnection between the two systems.

### Trigeminovagal connections described in animals

In 1966, Rhoton *et al*. reported that some vagus afferent fibers in Cynomolgus monkeys can turn caudally in their course over or through the Sp5 and *sp5* into the dorsal part of the Sp5 and *sp5*. This minority of fibers could be followed more caudally up to spinal cord level C3, where they terminated in the dorsal horn. In its descent, the vagus fibers give off both ventrally and dorsally directed fibers. The ventral fibers were observed to end about the cells of the interpolar part of the Sp5. The dorsally directed fibers were seen to disperse in the cuneate nuclei^15^. Contreras *et al*. investigated the central projection of the trigeminal, facial, glossopharyngeal and vagus nerves in rats and found terminals of these cranial nerves within the nucleus of the solitary tract^16^. Kishida *et al*. demonstrated that primary vagus C-fibers terminated in the lateral descending trigeminal complex (lateral descending trigeminal nucleus and tract), in crotaline snakes^17^. Similarly, Arends & Dubbeldam noted that vagus afferents in the mallard projected to the principal sensory nucleus of the trigeminal nerve and descending trigeminal tract^18^. In a study conducted by Brahic *et al*., which was carried out in frogs, labeled cells and terminal fields of the glossopharyngeal-vagal complex were found, amongst other sites, in the contralateral motor nucleus of the trigeminal nerve^19^. Equivalently in frogs, Kecskes *et al*. discovered that axon collaterals from the spinal- and mesencephalic tract of the trigeminal nerve could be followed into the glossopharyngeal-vagal motor neurons, alongside the perikaryia and dendrites of the nucleus ambiguus (Amb)^20^.

A similar anatomical interconnectivity found in the brains of the aforementioned species could also be present in humans. Towards investigating such an interactive trigeminovagal complex in humans, multiple, advanced neuroimaging techniques (i.e., 11.7T *ex-vivo* magnetic resonance imaging (MRI) and polarized light imaging (PLI) microscopy) should be used. PLI is a microscopy technique that quantifies fiber orientation based upon birefringence of the myelin sheath in histological brain sections and has been reported as a technique that is highly effective in validating MR findings^21^. The aim of this study was to combine post-mortem, 11.7T MRI, and PLI results in order to demonstrate the existence of the trigeminovagal complex in humans.

## Materials and Methods

Acquisition of specimens and detailed information on how PLI microscopy and MRI data from *ex vivo* brain tissue were acquired and processed was described previously in detail^22^.

### Included specimens and preparation

Four brains were retrieved from the body donor program of the Radboud university medical center (Radboudumc, Nijmegen, The Netherlands). All body donors in this program had signed a written informed consent during their lifetime permitting the use of their body and parts for science and teaching. The body donor program of the Radboud University Medical Center was approved by the National Medical Ethical Committee of the Netherlands and was legislated under Dutch law. Furthermore, this study and its applied methodology was performed under the approval of the Medical Ethical Committee of the Arnhem–Nijmegen region in the Netherlands. In addition, all methods were performed in accordance with the Declaration of Helsinki. The included donors had no known neurological diseases and none of the brains showed pathological deformities, macroscopically or microscopically.

All specimens were fixed within a short post-mortem interval (24-hours after death) through arterial embalming via the femoral artery with 10% formaldehyde. This is known to limit the reduction of the apparent diffusion coefficient (ADC) and fractional anisotropy (FA)^23,24^, two parameters that are known to be of crucial importance in diffusion weighted MR imaging. Following the embalmment, the specimens were immersed in 7.7% formaldehyde for 3 months- three years^25^.

Subsequently, each brain was extracted from the skull, and the brainstem and cerebellum were separated from the cerebrum by a transverse section, perpendicular to the neural axis at the level of the cerebral peduncle, rostral to the superior colliculi. The cerebellum was dissected from the brainstem by placing a section through the middle cerebellar peduncle. After this, the medulla was separated from the pons by a transverse section, perpendicular to the neural axis. All parts were stored in a container filled with 7.7% formaldehyde and preserved for an additional two months. See Table 1 for additional information on the specimens used in this study.

**Table 1:** Characteristics of the specimens used in this study.

Characteristics	Specimen #1	Specimen #2	Specimen #3	Specimen #4
*Age*	72 years	82 years	62 years	67 years
*Gender*	Male	Female	Male	Male
*Cause of death*	Colon cancer	Cardiac arrest/Congestive heart failure	Lung carcinoma	Cardiac arrest/Congestive heart failure
Time between death and fixation*	11 hours	16 hours	15 hours	16 hours
*Time between fixation and extraction of the brain*	2 months	3 months	2 months	4 months
*Time between extraction of the brain and scanning*	5 months	N/A	N/A	N/A
*11*.*7T*	Yes	No	No	No
*PLI*	Yes	Yes	Yes	Yes

PLI = polarized light imaging; * = post-mortem interval

### Magnetic resonance image acquisition

Prior to MR-scanning, the medulla of specimen #1 was soaked in a phosphate-buffered saline solution (PBS 0.1M, pH 7.4) for five days so as to reverse the decreased T2 relaxation rate induced by formaldehyde fixation^26^. Then, the medulla was placed for 24 hours in a 100 ml syringe, filled with a susceptibility-matched, hydrogen-free liquid (Perfluoropolyether, Fomblin®, *Solvay Solexis Inc*). Imaging was performed on an 11.7T Bruker BioSpec Avance III preclinical MR system (*Bruker BioSpin*, *Ettlingen*, *Germany*) equipped with an actively shielded gradient set of 600mT/m (slew rate 4570 T/m/sec). A circular polarized resonator was used for signal transmission and an actively-decoupled birdcage coil (*Bruker BioSpin*, *Ettlingen*, *Germany*) was used for receiving. Scanning was performed at 20 °C.

T1 FLASH weighted images were acquired using a 3D multi-gradient echo sequence at 0.2 mm isotropic resolution. TR = 25 ms; TE = 3.4 ms and a Flip angle = 10° were applied. The chosen parameters and applied MR protocol was adapted from an empirically designed protocol reported in the literature^27^ and has been reported previously by our group^22^. dMRI data were obtained using a segmented spin-echo with echo-planar imaging at 0.5 mm isotropic resolution. Four segments, covering a total of 256 gradient directions at a b-value of 4000 s/mm^2^, completed with six images with no diffusion weighting (b = 0 s/mm^2^) were used. Parameters included: Δ = 12.5 ms; δ = 4.0 ms; TR = 13.8 s; and TE = 30.7 ms.

### Track density imaging maps

Track density imaging (TDI) maps are typically used to investigate fiber structure at a resolution smaller than the imaging resolution. TDI uses reinterpolation of the quantitative maps by constrained spherical deconvolution based on the response function at a finer resolution^28^. TDI was applied to the raw dMRI data to study the medullar trajectories of the vagus nerve and its projections. To generate directionally-encoded color TDI maps, whole medulla probabilistic fiber-tracking was carried out with MRtrix^28^. A total number of 500,000 streamlines were generated (20 per seed voxel) from a large number of random seeds throughout the medulla. Seeds were placed randomly throughout the medulla as described before^28,29^. The total number of streamlines was calculated in each element of a grid that covers the medulla. The sampling distance of this grid was smaller than the acquired voxel size, yielding TDI map with higher spatial resolution than the resolution of the source dMRI data (0.1 mm isotropic). After tracking, the streamline density was used as intra-voxel information to construct a super-resolution TDI image^28^. Prior to creating the TDI map, the SIFT algorithm was plotted to the whole medulla fiber-tracking data by use of MRtrix. The SIFT algorithm was applied in order to match the streamline densities with the fiber orientation density lobe integrals as described by Smith *et al*.^30^.

### Histological tissue processing and polarized light imaging

Histological sectioning of the medulla of Specimens #1–4 was performed in order to optimally visualize all intramedullar fibers originating from the vagus nerve entry zone. Prior to sectioning, as cryoprotection, all specimens were immersed in a 30% sucrose-solution in 0.1M PBS at 4 °C for seven days. Each specimen was frozen by using dry ice and serially sectioned with a HM 450 Sliding Microtome *(Thermo Fisher Scientific Inc*., *Waltham*, *Massachusetts*, *USA)* at a slice thickness of 100 microns. In a series of three axial sections, the first section was mounted on glass, creating an inter-slice distance of 300 microns. All mounted sections were cover-slipped using the mounting medium, polyvinylpyrrolidone. After mounting and cover-slipping, the sections could be used for PLI microscopy.

The images obtained by PLI microscopy are based on the birefringent capacities of myelin that surrounds the axons. Therefore, PLI is especially suitable for visualizing the orientation of myelinated fibers and enables researchers to distinguish different fiber pathways in densely packed areas^21,31^. A Zeiss Axio Imager A2 microscope (*Carl Zeiss Microscopy LLC*, *United States*) was upgraded with a stationary polarizer, a quarter wave plate and a rotating polarizer and, thereafter, used as polarization microscope. In this set-up, white light first passes through the stationary polarizer and a quarter wave plate positioned in a 45° angle relative to the stationary polarizer to create circularly polarized light. Once polarized, the light passes through the specimen section containing birefringent myelin, which induces a phase shift. To capture the extent of this phase shift, nine images were acquired with a charge couple device camera at equidistant angles of the rotating polarizer from 0° to 180° (0–20°, 20–40°, 60–80° et cetera). Together with the 1.25x magnifying objective, this yielded a spatial resolution of approximately 4 μm/pixel. This polarization microscope set-up was used to scan a grid of high-resolution images. Slices were, therefore, divided into different field of views (FOVs) to cover the entire specimen. Only the green channel was used for further processing as the quarter wave plate was designed for this wavelength. A set of background images were acquired for every rotation angle to correct for inhomogeneous background illumination. Background correction of the images was performed as previously described by Dammers *et al*.^32^. Three different parameters were derived from the raw PLI data by fitting the light intensity at each pixel to a sinusoid: (1) the phase of the sinusoid, (2) the phase shift induced to the light wave and (3) the average amount of light passing through the tissue. Each of these parameters provided a different PLI map. Firstly, the phase of the sinusoid provided the in-plane orientation map. Secondly, the phase shift induced to the light wave provided the retardance map. Thirdly, the transmittance map was calculated as the average amount of light passing through the tissue. By combining the retardance-map and the in-plane orientation-map, the fiber orientation map was acquired, allowing different visualizations of the direction of myelinated fibers within the tissue^21^. All maps of the different FOVs were stitched to provide an overview of the entire slice by in-house written software in MATLAB^©^ (*The MathWorks, Inc. 1994–2017*).

Anatomical findings will be presented in agreement with the standardized Paxinos-Watson abbreviation system, which has been adopted by the majority of the available anatomical atlases, including the atlases of Paxinos and Huang^33^ and Mai *et al*.^34^.

## Results

### T1 FLASH- and dMR images and TDI maps

**Figure 1:**
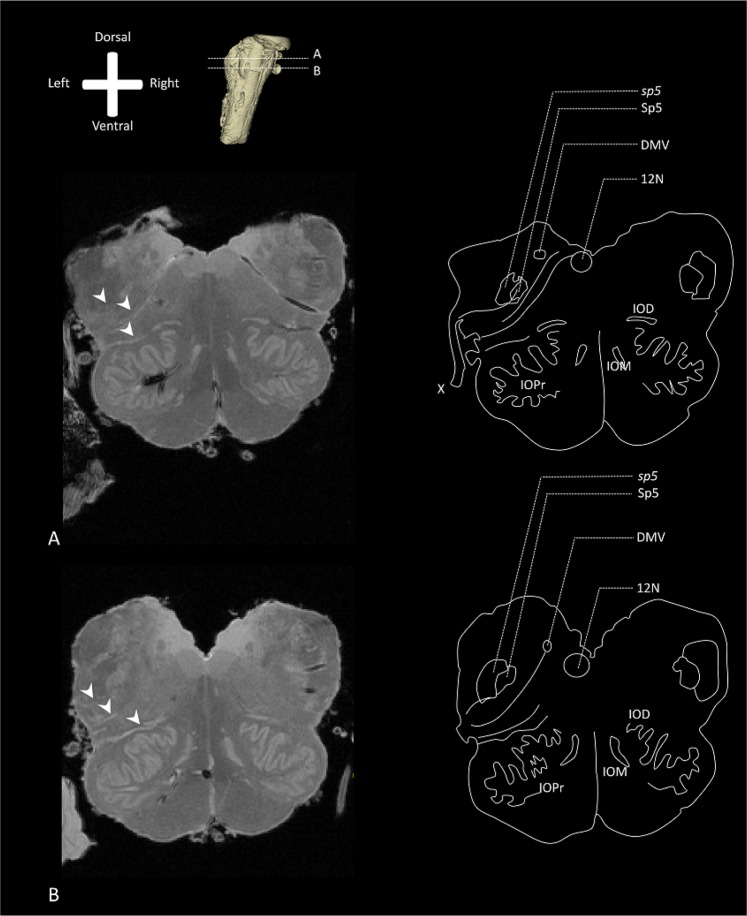
Overview of the transverse T1 FLASH MR images of the human medulla (Specimen #1). Anatomical orientation depicted by the anatomical anemone in the above-left corner. Next to the anemone, a three-dimensional model of the medulla depicts the level of transection. IOD: Dorsal inferior olivary nucleus; DMV: Dorsal motor nucleus of the vagus nerve; 12N: Hypoglossal nucleus; IOM: Medial inferior olivary nucleus; IOPr: Principal inferior olivary nucleus. (**A**) Superior transverse section through the rostral medulla depicting vagus medullar fibers (white arrow). Various anatomical landmarks can be recognized and are depicted in the schematic drawing on the right. (**B**) Inferior transverse section through the rostral medulla depicting vagus medullar fibers (white arrow). Various anatomical landmarks can be recognized and are depicted in the schematic drawing on the right.

The 11.7T MR images show that on the ventral part of the medulla, the pyramids can be distinguished, containing the pyramidal tracts of each hemisphere. Just more dorsally, the principal inferior olivary nucleus (IOPr) can be observed as extending to the ventrolateral surface of the medulla, bulging towards the lateral surface, causing the olive on each side of the brainstem. More medial to the IOPr, the medial inferior olivary nucleus (IOM) can be distinguished, located just lateral to the rostral ventromedial medulla and raphe. Immediately dorsolateral to the olive on each side, the vagus nerve can be observed entering the lateral part of the rostral medulla. Within the rostral medulla, these medullar fibers of the vagus nerve can be observed as hyper-intense striae. The hyperintense striae can be seen to cross a hyperintense area in the lateral medulla, at which the *sp5* and Sp5 can be located. The vagus pathways, furthermore, can be observed coursing towards the dorsal part of the medulla, also known as the area postrema. The area postrema contains the dorsal motor nucleus of the vagus nerve (DMV), the nucleus of the solitary tract (Sol) and the hypoglossal nucleus (12N). The DMV and 12N are both distinguishable on the acquired transverse T1-FLASH MR-images (Fig. 1).

**Figure 2:**
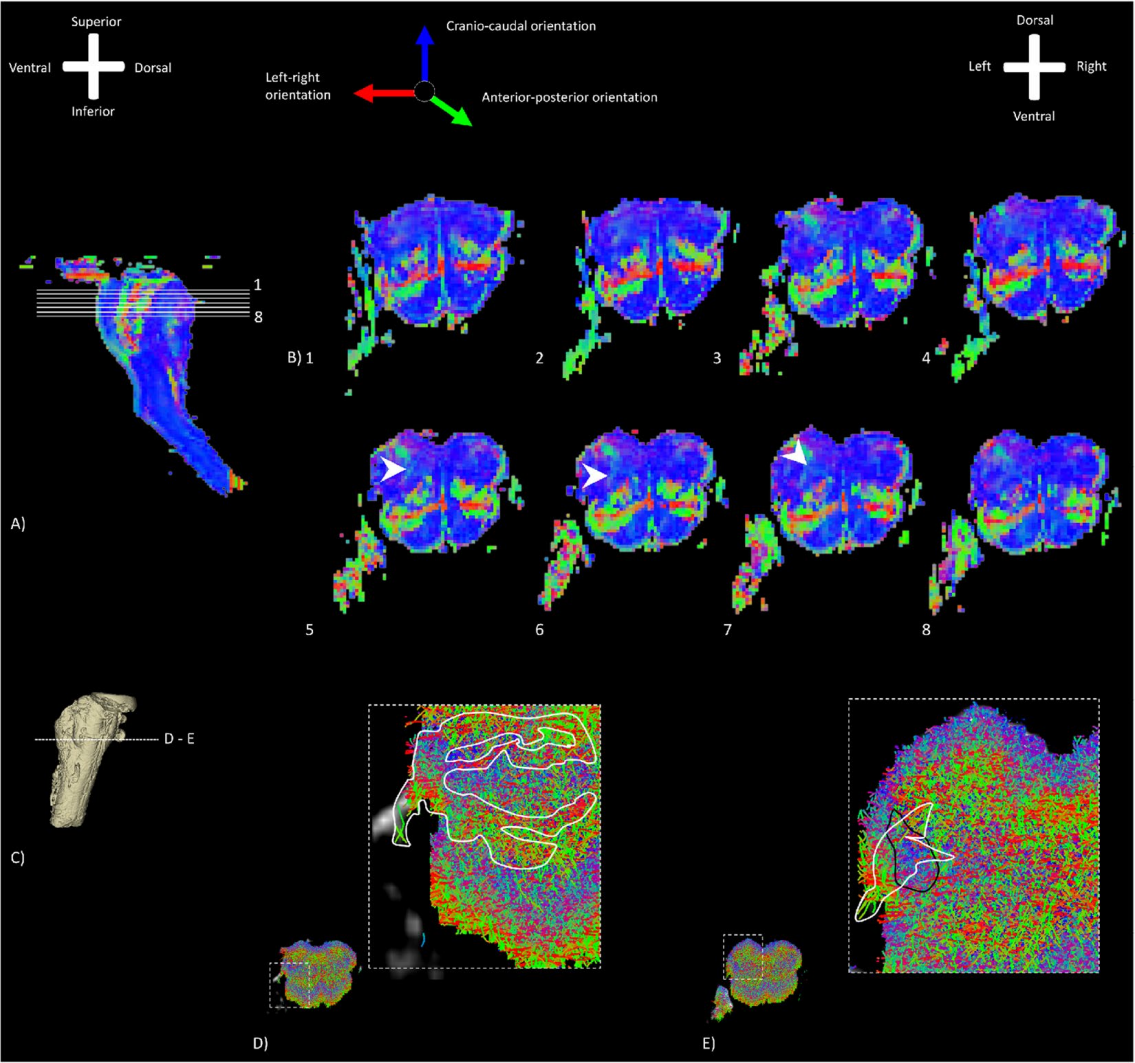
Series of color-coded dMR images of the rostral medulla (Specimen #1). The RGB (red–green–blue) color cross indicates the principal eigenvector orientations, red = left–right, green = anterior–posterior, blue = cranial–caudal. Anterior-posterior oriented voxels can be observed which form striae in the lateral medulla, probably representing the vagus medullar fibers (white arrow). (**A**) Midsagittal image depicting the levels of the transverse sections displayed in the second part of this image. Anatomical orientation depicted by the anatomical anemone in the above-left corner. (**B**) Series of transverse sections through the rostral medulla depicting the medullar fibers at different levels. Anatomical orientation depicted by the anatomical anemone in the above-right corner. (**C**) Three-dimensional model of the medulla depicting the level of transection. (**D**) Detailed TDI map of the medullar fibers sprouting from the vagus nerve entry zone, diverging into the lateral medulla. The fiber density is encoded by brightness. The fiber orientation is represented by color, while the fiber density is encoded by brightness. (**E**) Detailed TDI map showing the intersecting of the vagus medullar fibers (encircled in white) with the s*p5* (encircled in black).

Figure 2 depicts a series of color-coded 11.7T dMR- and TDI images (red-green-blue coding, indicating the left-right-, anterior-posterior- and cranio-caudal orientation of voxels, respectively) of the medulla. Small green striae can be observed in the lateral medulla, originating from the vagus nerve entry zone (Fig. 2B). These anterior-posterior-oriented striae on the dMR images are consistent with the hyperintense striae observed on the T1 FLASH images. Starting and termination points of these striae are, nevertheless, difficult to define due to the surrounding white matter structures. These large cranio-caudally oriented tracts that ascend and descend within the medulla^35^ dominate the dMR images. On the TDI maps, the vagus nerve and the vagus nerve entry zone can be recognized (Fig. 2). The vagus striae which were described on the T1 FLASH- and dMR images can be distinguished in the densely packed fiber pattern that constitutes the lateral medulla. Contrary to the dMR images, the TDI maps do show a variety of left-right oriented and anterior-posterior oriented fiber tracts sprouting from the vagus nerve entry zone (Fig. 2D). TDI maps, furthermore, show clearly that a densely-packed, cranio-caudally oriented fiber tract intersects the vagus nerve entry zone in the lateral medulla. This tract in the lateral medulla is recognized, as the spinal trigeminal tract (*sp5*) (Fig. 2E).

### Polarized light imaging microscopy images

**Figure 3:**
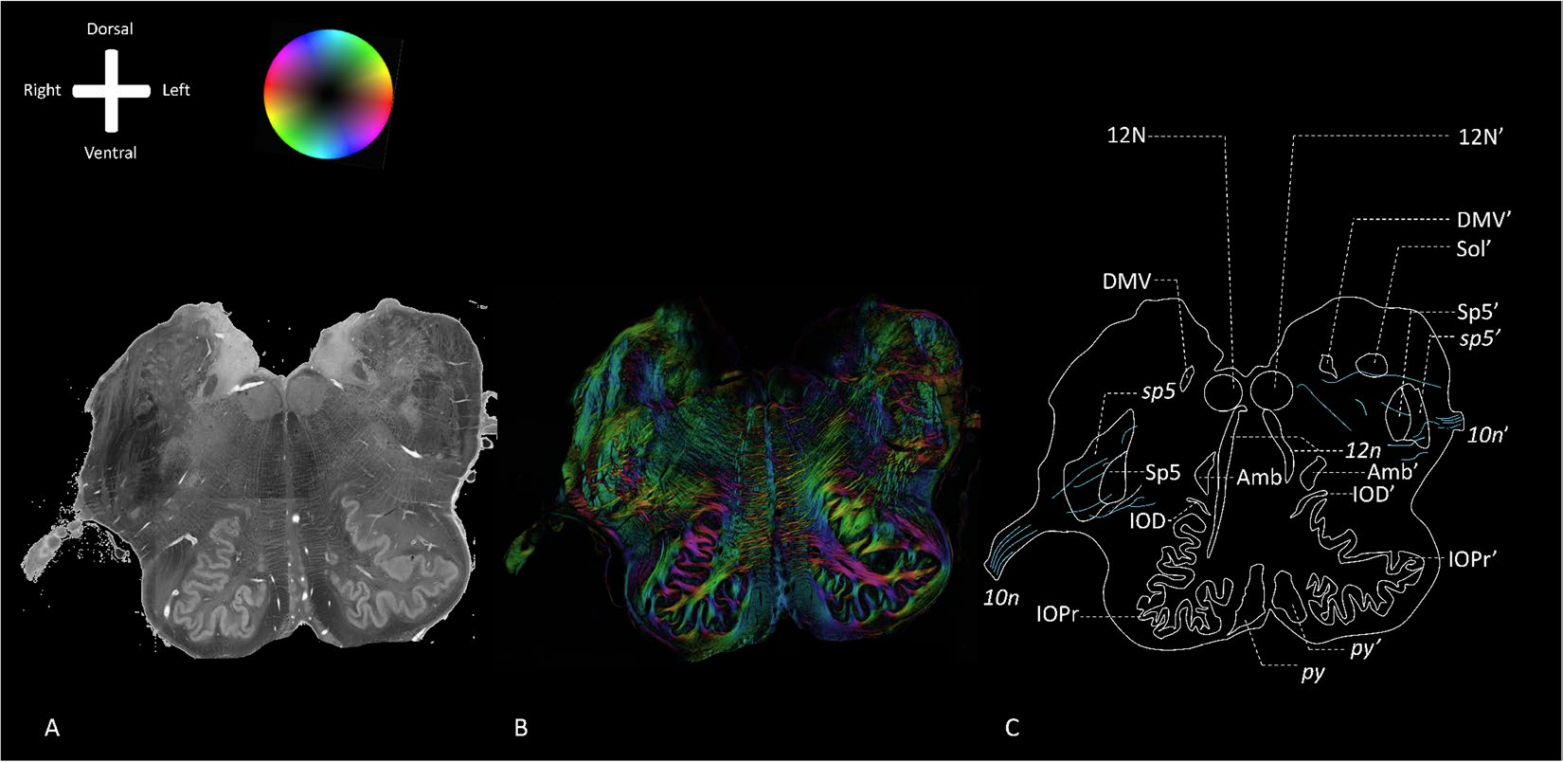
Polarized light imaging of specimen #1 at the level of the vagus root entry zone (Specimen #1). Anatomical orientation depicted by the anatomical anemone in the above-left corner. The fiber orientation is defined by the color sphere in the upper left corner. IOD: Dorsal inferior olivary nucleus; DMV: Dorsal motor nucleus of the vagus nerve; 12N: Hypoglossal nucleus; *12n*: hypoglossal nerve; IOM: Medial inferior olivary nucleus; Amb: Nucleus ambiguus; Sol: Nucleus of the solitary tract; IOPr: Principal inferior olivary nucleus; *py*: Pyramidal tract; Sp5: Trigeminal spinal nucleus; *sp5*: Trigeminal spinal tract; *10n*: Vagus nerve; Caudal pons*: Due to sectioning, the caudal pons and cerebral peduncle were deflected downwards, creating a partially diagonal transverse section through the caudal pons and the inferior olivary nucleus complex; ‘: same structure as on the right, which is depicted on the left. (**A**) Transmittance image of a transverse section at the level of the vagus root entry zone in the rostral medulla. (**B**) Color-coded fiber orientation image of a transverse section at the level of the vagus root entry zone in the rostral medulla. (**C**) Schematic drawing of a transverse section at the level of the vagus root entry zone in the rostral medulla.

**Figure 4:**
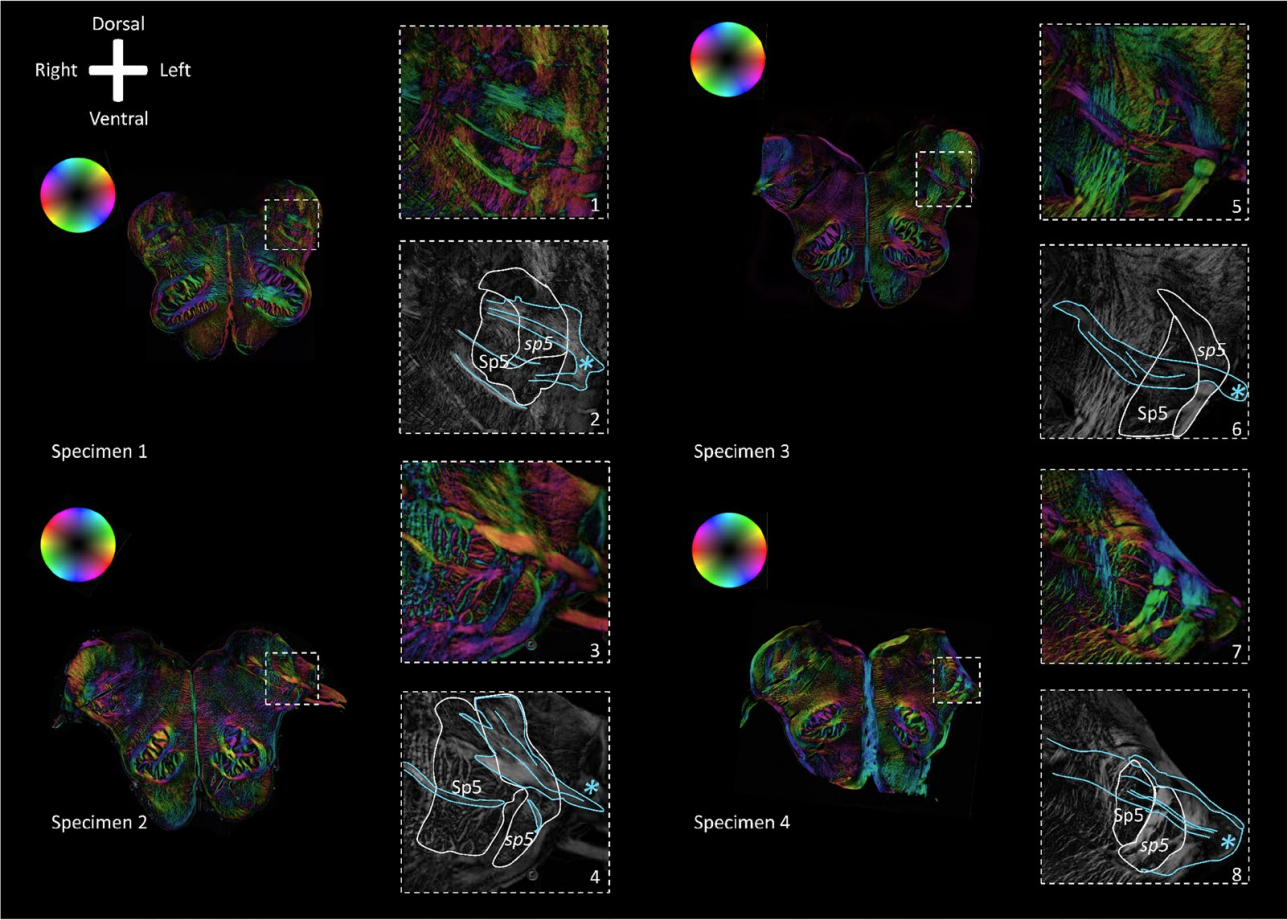
Polarized light imaging images of the four specimens at the level of the intersection of the *sp5* with the vagus fibers. Anatomical orientation depicted by the anatomical anemone in the above-left corner. The fiber orientation is defined by the color sphere in the upper left corner. Even numbers represent color-coded fiber orientation images. Odd numbers represent the corresponding transmittance images. Sp5: Trigeminal spinal nucleus; *sp5*: Trigeminal spinal tract. In all four specimens, the vagus fibers can be observed intersecting the *sp5* and Sp5. In specimen #1, #2 and #4, vagus fibers can be distinguished sprouting of the vagus trajectory to join the *sp5* and Sp5.

**Figure 5:**
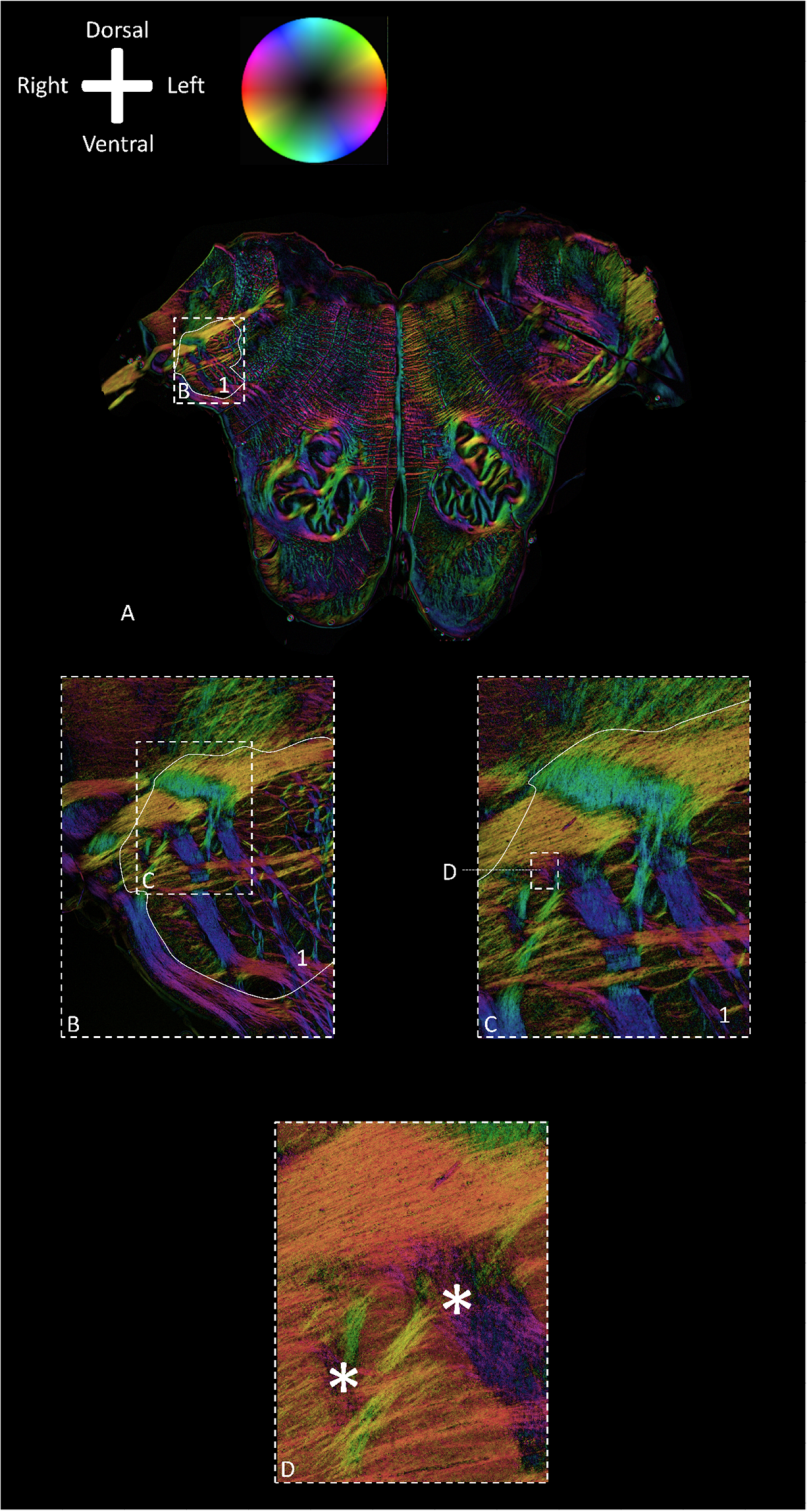
Polarized light imaging images of the vagus medullar fibers at different magnifications (specimen #2). Anatomical orientation depicted by the anatomical anemone in the above-left corner. The fiber orientation is defined by the color sphere in the upper left corner. Sp5: Trigeminal spinal nucleus; *sp5*: Trigeminal spinal tract; Delineated area 1 contains both the Sp5 (medial portion) and *sp5* (lateral portion). (**A**) Magnification 1x; (**B**) Magnification 2.5x; (**C**) Magnification 5x; (**D**) Magnification 10x At different magnifications, the vagus tract can be seen (yellow) to course towards the area postrema of the brainstem. From the vagus tract, fibers can be seen to change orientation (with associated color changes from yellow to orange and purple), depicting the sprouting of vagus fibers within the *sp5* and Sp5 (delineated by white lines; *sp5 is* located lateral, Sp5 is a more medially located structure). Merging of the vagus and trigeminal system can be seen at the asterisk (*) in Figure D. Vagus trajectory (orange) can be observed within the Sp5 and *sp5*. The vagus fibers entwine with trigeminal fibers (yellow and purple entwinement at the asterisks).

In the transverse sections of the rostral medulla, the IOPr can be identified as an important medullar landmark. The IOPr can be recognized as an undulating, thin sheath of grey matter, located just dorsal to the pyramids, containing the pyramidal tracts. Medial to the IOPr, the hypoglossal fibers can be distinguished, coursing just medial to the raphe, with an anterolateral orientation. The hypoglossal tract can be followed to the dorsal aspect of the brainstem, the area postrema in particular, up until the hypoglossal nucleus. Lateral to the hypoglossal tract and dorsal to the IOPr, the Amb and the constituents of the trigeminal medullar complex (i.e. the spinal trigeminal nucleus (Sp5) and *sp5*) can be distinguished (Fig. 3**)**. Just lateral to the Amb, Sp5 and *sp5* and dorsal to the IOPr, the vagus nerve enters the lateral rostral medulla in between the olive and the lateral funiculus as the vagus nerve entry zone. From there, vagus fibers can be followed to the center of the medulla adjacent to the obex, where the DMV and Sol can be found (Fig. 3). Figure 4 provides a more detailed overview of a consecutive series of PLI images of all specimens. This series shows that vagus medullar fibers course in between the white matter tracts of the lateral medulla and disperse into two directions: (1) towards the Amb and; (2) towards the DMV. In the lateral medulla, the vagus medullar fibers can be seen to intersect the Sp5 and *sp5* in all specimens (Fig. 4). When this region of interest was studied at higher magnifications, more detailed PLI images show fibers separating from the vagus trajectory. These sprouting vagus fibers disperse in a ventromedial direction and can be seen coursing in between the trigeminal fibers within the *sp5* (Fig. 5). Finally, the two systems can be seen to merge as the origins of the fibers become undistinguishable.

## Discussion

This study is, to the authors’ knowledge, the first to report on the trigeminovagal complex in man by use of 11.7T post-mortem MRI and PLI. This insight contributes to the investigation of experimental VNS as a treatment of primary headache disorders. It could, furthermore, lead to inclusion of other head- and facial pain conditions, like trigeminal neuralgia and neuropathic orofacial pain, to be treated with VNS.

### Structural research on the trigeminovagal complex

As this study shows, the vagus- and trigeminal systems are entwined at the level of the Sp5 and *sp5* in humans. These results are in agreement with anatomical evidence from animal-based research. In 1985, Gwyn *et al*. reported afferent projections to the ipsilateral Sp5 after horseradish peroxidase injection into the vagus nerve in the squirrel monkey^36^. Similar results were found in the cat using a similar methodology^37^. An oscillographic study confirmed results that the vagus nerve has central connections with the Sp5and the *sp5* in the cat^38^. Autoradiographic investigation showed that ascending fibers of the vagus nerve project to the Sp5 and the caudal pole of the principal sensory nucleus of the trigeminal nerve in monkeys^39^. The Sp5 can be separated into three sub nuclei (i.e., the caudal-, interpolar- and caudal subnucleus) based on its functional connectivity and cyto-architecture^40–47^. Based on connectivity, the caudal subnucleus of the Sp5 is known to play a central role in the conduction of nociceptive input from the orofacial region^47^. These findings are also observed in clinical settings from medullary trigeminal tractotomy (Sjöquist procedure)^48^. The caudal subnucleus extends from the level of the obex to the third cervical cord level^41^ and is known to have efferents to the ipsilateral- and contralateral thalamus (the mediodorsal nucleus and ventral posteromedial nucleus in particular)^49–51^, the periaqueductal grey^52,53^. The importance of the anatomical connections of the different vagus nuclei and their different subnuclei in treating primary headaches with VNS has been described by our group in a previous publication^54^. Based on these findings, the authors hypothesize that the majority of vagus fibers merge with the Sp5 at the level of the caudal subnucleus. Although difficult to distinguish from the interpolar subnucleus (rostral border) and cervical dorsal horn (caudal border), the caudal subnucleus has been reported to extend from the level of the obex to the third cervical cord level. At the same level, vagus fibers enter the lateral medulla and, according to the present study, join the Sp5 and *sp5*.

### Functional research with regard to trigeminovagal connections

Based upon the results from the present anatomical work, the authors hypothesize that structural connections between the Sp5 and s*p5* form the relay system, on which VNS in treating primary headache disorders depends. However, one of the major limitations of this research concerns that it cannot provide functional evidence of such a connection. Functional research in animals showed that electrical stimulation of the vagus nerve-induced expression of c-Fos like immunoreactivity, an indirect marker of neuronal activity, in the Sp5^55,56^. Other research in animals showed that a vagotomy produced a decrease in the expression of c-Fos-like immunoreactivity in the Sp5^57^. In humans, VNS was found to increase the connectivity between the nucleus of the solitary tract and the anterior insula and anterior midcingulate cortex. This increased connectivity was inversely correlated with time to the next migraine attack, suggesting clinical relevance to this change in connectivity^58^. The same group also presented new results in which they used high spatial resolution fMRI to investigate the brainstem response to VNS. Findings have shown that an increased response was seen to VNS in the olivary nucleus, Amb, Sp5 and the nucleus of the solitary tract^59^. Such functional changes induced by VNS could be explained by structural connections between the Sp5 and the vagus nerve system, as described in the present study. In preclinical research, VNS was found to inhibit trigeminal nociception in a rodent model of episodic migraine. To explain these results, the investigators showed inhibited activation of microglia and astrocytes in the Sp5 after VNS^60^. Another study found that VNS in rats elevated activity of neurons in the nucleus of the solitary tract. Other observations concerned the indirect activation of a subpopulation of second- and higher-order neurons, suggesting that afferent mechanisms and central neuron activation may be responsible for VNS efficacy^61^. Other functional evidence of a trigeminovagal complex in humans is derived from observations that VNS can modulate the cranial trigeminal autonomic reflex^62–64^. The existence of trigeminovagal connections have also been hypothesized based on findings from trigeminal nerve stimulation. Indeed, trigeminal nerve stimulation can modulate nociceptive signals in the trigeminocervical complex via activation of the vagus system^65–68^.

### Strengths and limitations

The most important merit of the present work concerns the fact that it shows, for the first time, that trigeminovagal connections exist in humans, whereas this has only been reported to exist in various animals. By combining 11.7T structural and diffusion MRI with PLI microscopy, the imaging resolution acquired by our images bridged the meso- to microscale (0.1 mm–5.0 mm)^69^, which is regarded as another strength of the present study. The absence of tractography data can be regarded as a shortcoming of this study. Nevertheless, it is known that tractography results can produce false-positive bundles of white matter anatomy^70^. Therefore, the authors aimed to provide dMRI information by use of the mean of the color-coded diffusivity and TDI-maps. Besides, PLI microscopy is known as a more suitable method for the visualization of white-matter anatomy^22,71^.

Various other imaging techniques were not implemented within this study design, forming another limitation. For instance, optical coherence tomography (OCT) yielding high resolution fiber architecture data with a spatial resolution of approximately 1 micron^72^, was not applied. Another technique that was not used in this study protocol is 3D-PLI, which provides the opportunity to investigate fiber orientations and their three-dimensional properties after sectioning^31^.Finally, more traditional techniques such as immunohistochemical staining and tracer techniques were not used in this study, as regular stains do not provide information about fiber orientations and tracers studies are rarely performed in humans. Nonetheless, Seehaus *et al*. showed that tracing studies can be performed in a post-mortem setting on adult human neural tissue over short trajectories with a maximal length of 13 mm^73^. The limited length of the trajectory made this technique unsuitable for tracing studies in humans. The same study, however, reported that DTI is capable of reflecting the shape and orientation of nerve pathways. Another limitation of this study is that it does not investigate or elucidate different patterns of vagus fibers merging with the Sp5 and *sp5*. A final limitation of this study is formed by the fact that it was not proved that vagus fibers synapse within the Sp5 as these applied techniques showed entwinement of the vagus fibers with the trigeminal fibers. Therefore, we cannot yet confirm that the neural trigeminovagal complex observed in this study also will be found at a synaptical level, although this has been proposed by various animal-based studies before. Future studies should, therefore, focus on the synaptical connections between the trigeminal- and vagus systems.

## Conclusion

This study shows that the trigeminal- and vagus systems interconnect anatomically at the level of the rostral medulla where the vagus fibers intersect with the Sp5 and *sp5*. These findings could aid the explanation of clinical observations and research, which found a functional connection between the trigeminal system and vagus system.
